# San Angelo District Medical Society

**Published:** 1902-11

**Authors:** L. C. G. Buchanan

**Affiliations:** Secretary


### San Angelo District Medical Society.

*Texas Medical Journal, Austin, Texas.*

DEAR DOCTOR DANIEL: The 14th inst. marked the first anniversary of the organization of the San Angelo District Medical Society.

We close a most profitable year's work and believe that we have one of the best societies in the State.

The newly elected officers are: President, Dr. F. B. Magruder; Secretary, Dr. L. C. G. Buchanan; Treasurer, Dr. C. T. Cooper.

Success to the "Red-Back."

Fraternally yours,

L. C. G. BUCHANAN,
Secretary.

